# Explaining differential item functioning focusing on the crucial role of external information – an example from the measurement of adolescent mental health

**DOI:** 10.1186/s12874-019-0828-3

**Published:** 2019-09-05

**Authors:** Curt Hagquist

**Affiliations:** Centre for Research on Child and Adolescent Mental Health, Karlstad University, SE-651 88 Karlstad, Sweden

**Keywords:** Differential item functioning (DIF), Resolving for DIF, Psychosomatic problems, Rasch model, Source of DIF, Validity and reliability, HBSC

## Abstract

**Background:**

An overarching objective in research comparing different sample groups is to ensure that the reported differences in outcomes are not affected by differences between groups in the functioning of the measurement instruments, i.e. the items have to work in the same way for the different sample groups to be compared. Lack of invariance across sample groups are commonly called Differential Item Functioning (DIF).

There is a sense in which the DIF of an item can be taken account of by resolving (splitting) the item into group specific items, rather than deleting the item. Resolving improves fit, retains the reliability and content provided by the item, and compensates for the DIF in estimation of person parameters on the scale of the instrument. However, it destroys invariance of the item’s parameter value among the groups. Whether or not a DIF item should be resolved depends on whether the source of the DIF is relevant or irrelevant for the content of the variable. The present paper shows how external information can be used to investigate if the gender DIF found in the item “Stomach ache” in a psychosomatic symptoms scale used among adolescents may reflect abdominal pain because of a biological factor, the girls’ menstrual periods.

**Methods:**

Swedish data from the international Health Behaviour in School-aged Children study (HBSC) collected in 2005/06, 2009/10 and 2013/14 were used, comprising a total of 18,983 students in grades 5, 7 and 9. A composite measure of eight items of psychosomatic problems was analysed for DIF with respect to gender and menstrual periods using the Rasch model.

**Results:**

The results support the hypothesis that the source of the gender DIF for the item “Stomach ache” is a gender specific biological factor. In that case the DIF should be resolved if the psychosomatic measure is not intended to tap information about abdominal pain caused by a gender specific biological factor. In contrast, if the measure is intended to tap such information, the DIF should not be resolved.

**Conclusions:**

The conceptualisation of the measure governs whether the item showing DIF should be resolved or not.

## Background

In a consensus paper published 20 years ago the International Epidemiological Association European Questionnaire Group brought up to front the deficiencies in measurement that were characterising epidemiology:

“*Epidemiological findings are often based partly or completely on responses to questionnaires. Yet the attention given to questionnaire development is often inadequate, compared with the amount of time and resources devoted to study design, population selection and analysing data.*” [1].

Clearly, progress has been made since then, but there are still many examples of epidemiological and public health studies conducted without sufficient examinations of the operating characteristics of the items of questionnaires that the analyses are based on. It follows that questions are frequently raised about the extent to which trends in self-reported mental health problems are distorted by deficiencies in measurement. Similarly, while there is overwhelming evidence of higher rates of internalising self-reported mental health problems among women than men, there are yet uncertainties to what extent these differences are affected by the methodology used for collecting and analysing the data.

These uncertainties and question marks are captured by the concept of invariance, which constitutes an inevitable part of comparative research across groups and time. To make valid comparisons across genders, a requirement for measurement is that the instruments work in an equivalent way for men and women. Violations of that requirement may distort the person measures and make the comparisons between genders invalid. Such requirements of invariant measurement were stated by Thurstone [2] already in the late 1920s and Guttman [3] in the 1950s, and formally specified in a mathematical model by Rasch [4] in the 1960s.

Lack of measurement invariance across sample groups was previously called item bias [5] but is nowadays commonly called Differential Item Functioning (DIF). The scale subjected to an analysis in the present paper consists of eight items. The persons are located along a variable (also referred to as trait or construct) according to their degree of psychosomatic problems measured as a summation of the responses across all items. DIF means that one or more items in a set of items, and for an equivalent psychosomatic problem, are functioning differently for different members of a sample group, e.g. for boys and girls. DIF may appear as uniform or non-uniform. If the magnitude of DIF is constant along the entire variable, i.e. regardless of the location of the person measures on the variable, then there is evidence of uniform DIF. In contrast, if the magnitude of DIF varies along the variable there is evidence of non-uniform DIF.

Over the years a large body of literature has been published on DIF, mainly about how to identify DIF but also on how to deal post-hoc with evidence of DIF. Different procedures available for detecting DIF are described by Osterlind and Everson [6] in a monograph exclusively dedicated to DIF. Their review covers the Mantel–Haenszel procedure, methods based on Item Response Theory and logistic regression, as well as some other methods [6]. Different methods to detect DIF seem to generate similar results. In a study examining DIF in two mental health scales all three methods applied (logistic regression, the Mantel-Haenszel procedure and Rasch analysis), showed consistent results [7].

In their DIF monograph Osterlind & Everson [6] highlighted a pitfall in conducting DIF analyses:

“*Sometimes, for reasons unknown, calculations of a DIF detection strategy may suggest DIF, where none truly exists*” (p. 21).

While similar observations were reported previously by other researchers, no explanations were given until the last few years when the concept of artificial DIF was introduced and artificial DIF was shown to be an artefact of the procedure for identifying DIF [8] whereby real DIF favouring one group in one item induces DIF in other items favouring the other group. In order to correctly interpret a DIF analysis, real DIF has to be distinguished from artificial DIF. If DIF items are misidentified, artificial DIF items may be wrongly deleted or resolved as if they were real DIF items. This in turn may negatively affect the properties of measurement.

In a paper Hagquist and Andrich [9] summarised recent advances of analysis of DIF and suggested a unified methodology, including the distinction and identification between real and artificial DIF.

A DIF analysis starts with examining the original item set for DIF across sample groups that are to be compared, followed by a sequential procedure for distinguishing real and artificial DIF items. Among the original items and for each sample group, the item showing the greatest DIF is considered to be a real DIF item and therefore resolved by splitting the item into group specific items (e.g. one for boys and one for girls if gender is subjected to the DIF analysis). The resulting item set including two group specific items is analysed. Following the resolution of an item, it cannot induce artificial DIF in other items, and once again the item, if any, showing the greatest magnitude of DIF is considered to be a real DIF item. If necessary, this sequential procedure continues in additional steps. Having identified the item(s) showing greatest real DIF, a decision has to be made whether to retain, resolve or remove the real DIF items in further analyses. Such a decision has to be accompanied and guided by external information, which is the topic of the present paper.

Ideally, when data fit the model of analysis, multiple items which assess different aspects of the same variable increase both the reliability and validity of measurement. In practice fit and invariance, reliability and validity, do not always coincide, implying situations where a trade-off between them needs to be considered. Improved fit is dealt with by resolving the DIF item into group-specific items implying that the fit of the data to the Rasch model may be improved and the reliability retained. The invariance of the item’s parameters is, however, compromised and the validity of the variable may be affected negatively. Therefore, Andrich and Hagquist [10] suggested a rationale for deciding whether or not resolving items which improves fit and retains reliability, but violates invariance while retaining validity, may be justified:

“*If the source of DIF can be understood as a result of an aspect irrelevant to the content of the variable and therefore deemed dispensable, then resolving the item and accounting for the DIF seem legitimate. However, if the source of DIF involves an aspect of the item relevant to the content of the variable and thus deemed indispensable, then resolving the item in a way that reduces the difference between the group means may seem dubious*.” (p. 202).

To illustrate the complexity of the trade-off between model fit and validity Hagquist and Andrich [9] provided an example of gender DIF from a psychosomatic problems scale for adolescents:

“…*But does this reasoning also apply when the gender DIF is likely to be caused by biological factors? For example, the DIF shown for the item Stomach ache in the present analyses may reflect abdominal pain because of the girls’ menstrual periods. It turns out that in dealing with this DIF a critical issue is whether this potential source of the DIF should be considered relevant or irrelevant for the conceptualisation of psychosomatic problems and its applications*.” (p. 7).

They concluded that without external information about the sources of the DIF it is difficult to decide whether to resolve and take account of a DIF item or not.

The present paper is capitalising on the previous work by Andrich and Hagquist [10] and Hagquist and Andrich [9] which was referred to above. Their suggested rationale for deciding whether or not resolving DIF items assumes that the source of DIF is known. Based on a tentative Rasch analysis of a psychosomatic symptoms scale used among adolescents they hypothesised that the DIF shown for the item Stomach ache may reflect abdominal pain because of the girls’ menstrual periods. This hypothesis will be further elaborated and tested empirically in the present paper using the Rasch model analysis of DIF. The purpose of the paper is to provide an illustrative and concrete example of how external information can be used for investigations of the sources of DIF.

## Methods

The study is based on data collected in the Health Behaviour in School-aged Children (HBSC) study among students 11, 13 and 15 years old [11]. The HBSC study currently includes 49 countries and regions across Europe and North America and is conducted in collaboration with the WHO Regional Office for Europe. Repeated data collections have taken place every fourth year since the 1980s. Data are collected in schools with a questionnaire which is completed anonymously in the classroom.

For the purpose of this study data from Sweden collected 2005/06, 2009/10 and 2013/14 are used. The entire data set used for this study comprises a total of 18,983 students; 6518 in grade 5, 6002 in grade 7 and 6463 in grade 9, corresponding to 11, 13 and 15 years old students. Across years, boys and girls as well as students in different grades were evenly distributed. At each year of investigation and grade the groups of students were homogeneous with respect to year of birth. The proportion of students belonging to the dominating year of birth varied between 93 and 97%.

The outcome measure subjected to the analysis in this paper is the HBSC checklist on psychosomatic symptoms, commonly called the HBSC-SCL, and frequently used in public health studies among adolescents [12]. This composite measure includes somatic as well as psychological complaints that are shown to reflect a common dimension. The term psychosomatic is used in a general sense, without making any presumptions about etiology [13].

The HBSC-SCL consists of eight items that follow the question “In the last 6 months, how often have you had the following complaints?”: Headache, Stomach ache, Backache, Feeling low, Irritability or bad temper, Feeling nervous, Difficulties in getting to sleep, Feeling dizzy.

The response categories for all of these eight items are ‘About every day’, ‘More than once a week’, ‘About once a week’, ‘About once a month’ and ‘Seldom or never’. The categories are ordered in terms of implied frequency and the higher frequency, the higher the degree of psychosomatic symptoms.

All DIF analyses of the psychosomatic scale were conducted using complete data, which reduced the sample size with 6.6%.

The following question about the menarche was included in the HBSC questionnaire:

“Have you begun to menstruate (have periods)?” with the response options yes or no.

DIF was analysed with respect to gender and the first menstrual period (menarche) among girls. Analyses of gender DIF conducted separately for each grade are briefly summarised. The period DIF is analysed at a finer level and reported in detail. Because the distribution of girls having had or not having had their first period was unevenly distributed among grades with some small sample groups within grades 5 and 9 the period DIF analyses were not conducted separately for each grade but based on the entire sample. The period DIF was analysed based on three sample groups: boys, girls not having had a first period and girls having had their first period. Because there were big differences in the sample size between these groups the sample size was randomly set to the value of 3500 for each group which was close to the original value for the smallest group. In the gender DIF analyses no adjustments were required because the groups of boys and girls were almost equally sized.

### The Rasch model and the analysis of Differential Item Functioning

The Rasch model is named after the Danish mathematician Georg Rasch and has invariance as an integral property [4]. Therefore, a test of the fit between the data and the model is a test of whether an instrument works invariantly across individuals and across sample groups. The Rasch model enables item and person parameters to be estimated independently of each other, which take the form of person and item location values placed on a common logit variable.

The polytomous Rasch model [14] takes the general form:

$$ \Pr \left\{{x}_{vi}=x\right\}=\frac{e^{-{\tau}_{1i}-{\tau}_{2i}\dots -{\tau}_{xi}+\left({\beta}_v-{\delta}_i\right)}}{\sum \limits_{x\hbox{'}=0}^{m_i}{e}^{-{\tau}_{1i}-{\tau}_{2i}\dots -{\tau}_{x\hbox{'}i}+x\hbox{'}\left({\beta}_v-{\delta}_i\right)}} $$

*β*_v_ is the location of person v and *δ*_i_ is the location of item i. *τ*_*xi*_; *x* = 1, 2, …*m*_*i*_ are thresholds which partioned the latent continuum of item *i* into *m*_*i*_ *+ 1* ordered categories. In the polytomous Rasch model [14] that has more than two categories the thresholds partition the latent continuum of each item into ordered categories. They are the points on the latent scale where the conditional probability of two adjacent categories is equal. Disordered thresholds may be an indication that the categorisation of an item does not work as intended [15]. Because the item thresholds appeared disordered in the Rasch analysis, two pairs of response categories (‘About every day’ & ‘More than once a week’ and ‘About once a week’ & ‘About once a month’) were collapsed based on analyses of the locations of the threshold values. This resulted in three response categories for each of the eight items [15]. The responses to the items were summarised and transformed into a linear logit scale on which higher values represented better health, and lower values worse health.

**Fig. 1:**
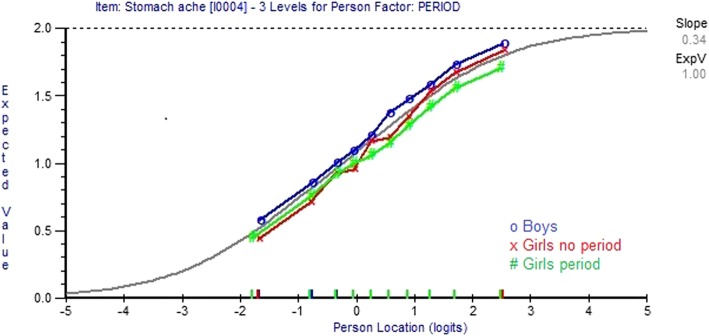
Item Stomach ache showing period DIF, in a set where item Felt low is resolved for period-DIF

Expected Value Curves (EVCs) predict the item scores as a function of the item parameters and person locations on the latent trait (see Fig. 1 for an example) [9]. If the observed means of persons in adjacent class intervals do not fit to the expected values of the curve there is lack of invariance. If an item functions differently for different members of a group, e.g. boys and girls, separate EVCs for each groups are required for an item. If this DIF is the same along the latent trait, i.e. if the EVCs are parallel, the DIF is referred to as uniform; if the DIF varies along the latent trait, i.e. the EVCs are non-parallel, DIF is referred to as non-uniform. No DIF means that the expected value of a response to an item is the same across genders for persons with the same value on the variable. The absence of DIF does not exclude the presence of gender difference in the frequencies of Stomach ache or other items between boys and girls or between girls having had their first period and those who have not had it. It simply means that there are no differences in item functioning across sample groups that contribute to differences in psychosomatic problems between for example boys and girls.

The DIF-analysis was conducted using a two-way analysis of variance of residuals based on parameters estimated by the Rasch model [9]. The ANOVA (Analysis of Variance) allows for simultaneously testing of uniform as well as non-uniform DIF among a priori specified sample groups. In that respect the ANOVA analyses the standardised residuals of responses from the estimated EVC. The ANOVA determines whether there is a main gender effect, a class interval effect, or an interaction between the class interval and gender.

Because the ANOVA as most procedures for identifying DIF induces artificial DIF, real and artificial DIF have to be distinguished [10]. In order to do that, items showing DIF have to be sequentially resolved starting with the item showing the worst DIF. The F-values calculated in the ANOVA give the rank order for each item corresponding to the magnitude of DIF.

Resolving an item showing evidence of real DIF means splitting the item into group specific items, e.g. one for boys and one for girls [10]. If the DIF is real, resolving an item will affect the difference in mean values between the groups, while artificial DIF will not have any such impact. Resolving a DIF item also enables the magnitude of the DIF to be quantified, by comparing the estimates of the item parameters (i.e. the location and slope values) from the different groups. When an item is resolved, responses for all groups except the designated group become structurally missing.

The DIF analysis is structured as follows:

First, the analysis of gender DIF for the item Stomach ache previously reported for grade 9 showing that girls are scoring such problems to a higher extent than expected by the Rasch model is expanded to also include students in grades 5 and 7. This is supposed to be an indirect test in order to examine if the hypothesis about the menarche as a possible cause of gender DIF is empirically justified. Based on existing knowledge about the menarche showing that only a small proportion of 11 years old girls (grade 5) have had their first period while almost all girls have had it at their age of 15 (grade 9) the magnitude of gender DIF is expected to be relatively small for Stomach ache in the grade 5, greater in grade 7, and even greater in grade 9.

Second, the hypothesis is directly tested by using information from the girls themselves about the age of their first menstrual period. Again from the hypothesis, DIF is expected to be clearly related to the menarche, with girls having had their first period scoring higher frequency on the Stomach ache question than those girls not having had a period, given that the girls who are compared (period vs no period) are located at the same place on the latent variable i.e. experiencing the same overall load of psychosomatic problems.

The Rasch model analysis was performed with the software RUMM2030 [16].

## Results

### Descriptive statistics on the menarche

In Table 1 the proportion of girls in grades 5, 7 and 9 who have had and not have had their first period is shown.

**Table 1:** The proportion of girls having had and not having had their first period, distributed by grade

	Grade 5	Grade 7	Grade 9
	*n* = 3174	*n* = 2929	*n* = 3168
Girls period	10.9%	70.7%	97.6%
Girls no period	89.1%	29.3%	2.4%

Table 1 shows that the proportion of girls having had their first period increases sharply across grades, with the biggest change from grade 5 to grade 7. While only one out of ten girls in grade 5 have had their first period, almost all girls in grade 9 have had it.

Table 2 shows the relative frequencies of Stomach ache among grade 5, 7 and 9 students, distributed by gender and menarche.

**Table 2:** Relative frequencies of Stomach ache among grade 5, 7 and 9 students, distributed by gender and menarche

	Boys	Girls no period	Girls period
	*n* = 9153	*n* = 3676	*n* = 5446
Every day	2.1%	3.7%	5.5%
More than once a week	5.5%	8.0%	13.6%
Once a week	11.3%	15.5%	16.8%
About once a month	29.0%	28.7%	44.0%
Seldom or never	52.2%	44.1%	20.0%

Table 2 shows that Stomach ache is more frequently reported by girls who have had their first period than among girls who have not had it as well as in comparison with boys. Among girls having had their period, more than four out of ten report having Stomach ache about once a month, while the proportions are less than 30% among the groups of boys and pre-period girls.

The differences between the two groups of girls are largest for the category seldom or never. The gender differences are also much smaller comparing boys with the pre-period girls than in comparisons with girls having had their first period.

### Categorisation of the items

Collapsing two pairs of response categories, (‘About every day’ & ‘More than once a week’, and ‘About once a week’ & ‘About once a month’) improved the overall item fit. Moreover, combining the two pairs of categories caused only a very small decrease of the person separation index (PSI), from 0.76670 to 0.76012. This is an indication that the original response set with five categories does not work properly. Since the number of data points was halved by the collapsing, a sharp drop of the PSI would have been expected if the original five response categories had worked properly.

### Gender DIF

Grade specific analysis of variance of the standardised residuals at the adjusted sample size of 1500 showed that Dizziness was the item with the largest magnitude of gender-DIF in grade 5. In grade 7 the items Felt low and Stomach ache showed roughly the same magnitude of gender DIF, indicated by similar F-values. In grade 9, Stomach ache was the item showing the largest magnitude of gender-DIF, followed by Felt low.

After resolving the item Dizziness, there was no gender DIF left in grade 5. In contrast, the gender-DIF was more evident in grade 7. Along the whole trait, girls are scoring lower values (=more frequent problems) than boys on the item stomach ache given the same location on the trait, and the DIF is uniform. This uniform DIF is further confirmed by a clear difference in item location values for boys and girls, but a very small difference in slope values. The patterns of gender DIF found in grade 7 is further pronounced in grade 9 with even bigger differences in item location values for boys and girls. DIF is also tending to be non-uniform. The DIF is greater for students with less overall psychosomatic problems.

### Period DIF

The analysis of variance of the standardised residuals at the adjusted sample size of 1500 showed evidence of period-DIF for three items. Among these, Felt low was the item with the highest F-value, i.e. largest magnitude of period-DIF. After splitting that item into three separate items, one for boys, one for girls not having had a period and one for girls having had their period, only the item Stomach ache showed significant DIF. Resolving also that item following the same procedure resulted in an item set with none of the remaining six items showing period-DIF.

**Fig. 2:**
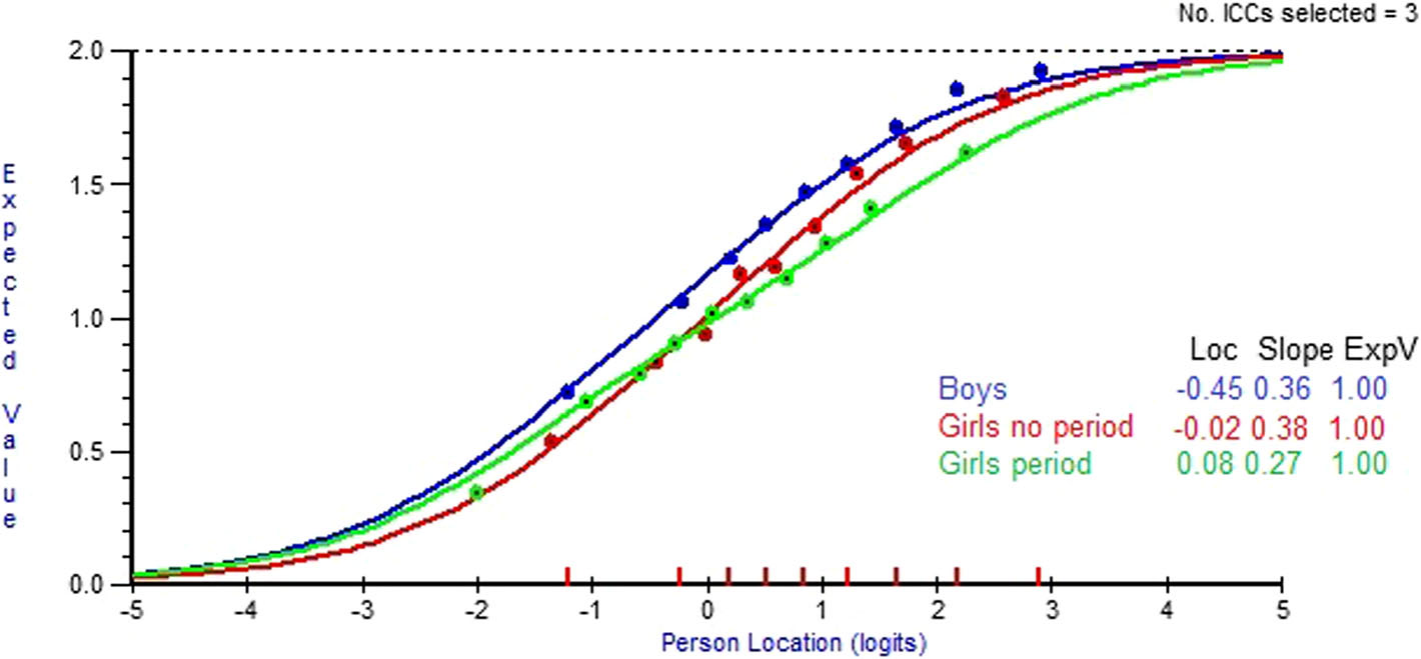
Item Stomach ache resolved for period-DIF, in a set where first item Felt low is resolved for period DIF

In Figures 1 and 2 the Expected Value Curves for the item Stomach ache are displayed in two different item sets showing period-DIF: a set where first Felt low has been resolved for period DIF (Fig. 1) and a set where both Felt low and Stomach ache have been resolved (Fig. 2).

Figures 1 and 2 show that there is evidence of period DIF. Among those with less overall psychosomatic problems, girls having had their first period are scoring lower (=more frequent problems) on the item Stomach ache than those not having had a first period. Using boys as a reference, at end of the trait with higher values the expected value curve for girls not having had a first period is closer to the curve for boys than for girls having had their first period.

Table 3 shows the person mean estimates of psychosomatic problems in the entire sample of students in grades 5, 7 and 9 for the original item set of eight items and for two revised item sets where the items Stomach ache and Felt low have been resolved for period DIF. In addition, overall item fit statistics are reported, along with the PSI, a measure of traditional reliability based on the Rasch model estimates of the person parameters and their standard errors.

**Table 3:** Mean values, item fit statistics and PSI values for three item sets among students in grades 5, 7 and 9. Randomly sampled group sizes of 3500

	Item Set 1 Original 8 items	Item Set 2 Resolving Felt low for boys; girls no period; girls period	Item Set 3 Resolving Felt low and Stomach ache for boys; girls no period; girls period	Difference in difference Set 2 and 3	Difference in difference Set 1 and 2	Difference in difference Set 1 and 3
Boys	1.088	1.065	1.042			
Girls no period	0.951	0.956	0.985			
Girls period	0.005	0.056	0.114			
Difference Boys-Girls no period	0.137	0.109	0.057	0.052	0.028	0.080
Difference Girls no period-Girls period	0.946	0.900	0.871	0.029	0.046	0.075
Person Separation Index	0.76195	0.75846	0.75501			
Overall item fit Chi Square Probability (adjusted sample size of 1500)	0.005361	0.182495	0.532964			

As expected from theory, the PSI values remain about the same after resolving the items “Stomach ache” and ‘Felt low’ as in the original item set. Also, the chi square probability values indicate a clear improvement of the overall item fit for each item that is resolved.

The patterns of the mean values for period DIF of item Stomach ache are consistent with patterns conveyed by the EVC curves shown in Figs. 1 and 2, i.e. the period DIF for item Stomach ache is confirmed by differences in person mean values before and after DIF has been resolved. While the differences in mean values between the period and no period groups of girls decreased, large differences in psychosomatic problems between the two groups remain also after the period DIF has been taking into account.

### Tentative analyses

The analysis of gender and period DIF was replicated tentatively on the original data set with items including five response categories showing disordered thresholds in the Rasch analysis. While the non-uniform DIF could be expected to be strengthened when distinguishing the categories ‘About once a week’ and ‘About once a month’ given the hypothesised source of the DIF, the outcomes rather showed the opposite, i.e. less evidence of non-uniform DIF. Considering the disordering of the thresholds indicating improper ordering of the response categories, this apparent counterintuitive pattern is not surprising.

## Discussion

DIF-analysis is an indispensable part of psychometric analyses aiming to investigations of invariant properties of a measurement instrument. In order to decide whether or not to resolve items showing evidence of DIF, information about the source of DIF is required. The purpose of the present paper was methodological: to provide an illustrative and concrete example of how external information, in this case about the menarche, can be used for investigations of the sources of DIF. The results presented in this paper support the hypothesis that the source of the gender DIF for the item Stomach ache included in a psychosomatic problems scale is likely to be girl’s menstruation, i.e. a gender specific biological factor. Both the gender DIF and the period DIF approaches used in this study point to the same conclusion. Separate DIF analyses in each of the three grades show that there is only a small gender DIF for the item Stomach ache in grade 5, i.e. at the age about 11 when only a small proportion of the girls have had their first period. In contrast, such DIF is more evident in grade 7 (about 13 years old) when a vast majority of the girls have experienced their first period and in grade 9 (15 years old) when almost all girls have had it. Hence, consistent with the hypothesis about the menstruation as a source of the DIF, the magnitude of the DIF is greater in grade 9 than in grade 7. While the gender DIF in grade 7 appears to be mainly uniform, the DIF in grade 9 tends to be non-uniform which means that the magnitude of the DIF varies along the trait. Among grade 9 students with lower degrees of psychosomatic problems the gender DIF is more pronounced than among students with higher degrees of such problems. The analysis including all three grades simultaneously clearly shows a period DIF, with girls who have had their first period reporting stomach ache most frequently. Among those students with lower load of overall psychosomatic problems the DIF between boys and girls not having had a first period is smaller than between girls who have had their first period and those who have not had it.

From a content perspective the DIF evident for the item Stomach ache seems explainable given the available knowledge about the age for the menarche. Given that, it’s not surprising that there is only a small gender DIF in grade 5 and that the strongest evidence of gender DIF is to be found in grade 9. The indicated interaction in grades 7 and 9 between period and gender respectively, and the class intervals along the trait, i.e. the non-uniform DIF, is also tentatively explainable. Among those students experiencing higher degrees of psychosomatic problems the relative impact of Stomach ache caused by the periods may be minor because of being part of an overall pattern of more frequent complaints.

The analysis of DIF reported in this paper also shows that additional information, which is external to the measurement instrument and outside the analysis which identified it, is required in order to investigate in depth the sources of the DIF. The access to data on the menarche enabled a direct test of a specific hypothesis, in addition to the descriptive DIF-analysis across grades. Because the hypothesis was supported, it follows that in a next step a decision has to be made whether the item Stomach ache should be resolved for gender DIF or not. Following the rationale quoted above which was suggested by Andrich and Hagquist [10], such a decision should not only be guided by statistical criteria but also by a consideration about the implications for the content of the measure. It should be noted that in group comparisons, resolving an item has the same consequences as removing an item, i.e. the impact of the resolved item on the group comparisons is taken away. Because the gender specific items for Stomach ache have different location and/or slope values, the estimates for the groups are no longer invariant.

While resolving an item may improve the fit of the data to the model and retain reliability it destroys invariance of the item parameters among the groups. Resolving an item may either improve or worsen the validity, depending on whether the source of the DIF is relevant for the content of the variable or not. If the source of the DIF is relevant to the measure, resolving an item may worsen the validity and vice versa.

If a measure subjected to a DIF analysis is clearly conceptualised, insights about the sources of the DIF may be sufficient in order to judge whether the source of the DIF is relevant to content of the variable in question and to decide whether the DIF should be resolved and taken account of or not. Because the concept of psychosomatic problems is not fully defined and there is not any presumption about etiology, knowledge about the sources of the DIF is necessary but not sufficient. It turns out that individual conceptualisations of the measure will govern whether the item showing DIF should be resolved or not. In the current example it needs to be considered if abdominal pain reflecting gender specific biological conditions should be a part of the measure of psychosomatic problems. If the measure conceptually is intended to tap information about Stomach ache regardless of causes, resolving the gender DIF, or removing the item, would negatively change the content of the variable. In contrast, if complaints caused by gender specific biological factors are not considered to be relevant for the measure of psychosomatic problems, resolving the item would not just retain the reliability and improve the fit of the data to the model but also improve the content validity of the variable. In the judgement of different options to handle the DIF, the available data may turn out to be insufficient to make a decision, requiring additional external information. It follows that the current paper doesn’t end up with a suggestion about which option would be best to choose in order to handle the DIF.

While the impact on the person measurement of the gender DIF for the item Stomach ache may seem relatively small, if there are other items showing DIF in the same direction, the overall effect on person measurement will certainly increase. Another item showing evidence of gender and period DIF of about the same magnitude and in the same direction as Stomach ache is the item Felt low.

## Conclusions

In conclusion, external information about the menarche was used to carry out an in depth analysis of the source of the gender DIF indicating that a biological factor was causing the gender DIF evident for the “item frequency Stomach ache”. The present paper also shows that in order to decide whether to resolve the DIF or not additional information may be required if the concept in question is not clearly defined. However, it may also be considered that regardless of the source of DIF, that invariance should take precedence over fit and that the item should not be resolved.

Finally, some remarks are in order. As stated above, the purpose of the present paper was methodological. The available external data on the menarche worked properly to provide an illustrative and concrete example of how external information can be used for investigations of the sources of DIF. While the psychosomatic problems scale is considered causal (reflective) [17–19] in that the degree of problems governs the probability of certain responses to each item and the analysis support the hypothesis about the menarche as a cause of the gender DIF, further quantitative and qualitative information is required in order to make firm conclusions about the causes of the gender DIF and to exclude alternative hypotheses. Also, additional data are required to examine possible interactions between period DIF and other variables, e.g. grades (age) as well as to identify possible interactions with year of investigations.

## Data Availability

The data were provided by the Public Health Agency of Sweden. Requests for data should be submitted to the Public Health Agency.

## Abbreviations

ANOVA: Analysis of Variance
DIF: Differential Item Functioning
EVC: Expected Value Curve
HBSC: Health Behaviour in School-aged Children
HBSC-SCL: HBSC symptom checklist
PSI: Person Separation Index
